# Chromosomal evolution and speciation inferred from chromosome-scale genome assemblies in three Cupressaceae species

**DOI:** 10.1093/dnares/dsag010

**Published:** 2026-08-04

**Authors:** Kenta Shirasawa, Yuta B Aoyagi, Kentaro Mishima, Hideki Hirakawa, Tomonori Hirao

**Affiliations:** Department of Frontier Research and Development, Kazusa DNA Research Institute, Chiba 292-0818, Japan; Department of Frontier Research and Development, Kazusa DNA Research Institute, Chiba 292-0818, Japan; Kyushu Regional Breeding Office, Forest Tree Breeding Center, Forestry and Forest Products Research Institute, Forest Research and Management Organization, Kumamoto 861-1102, Japan; Faculty of Agriculture, Kyushu University, Fukuoka 819-0395, Japan; Forest Tree Breeding Center, Forestry and Forest Products Research Institute, Forest Research and Management Organization, Ibaraki 319-1301, Japan

**Keywords:** chromosome-scale genome assembly, comparative genomics, Cupressaceae, forestry, genetic mapping

## Abstract

Chromosome-scale genome assemblies in gymnosperms have lagged behind those of angiosperms, likely due to their large genomes. Coniferous tree species, which belong to the gymnosperms, are important resources for wood production in the forestry industry. To elucidate the evolution and speciation of these species and establish genome resources for breeding, we integrated draft assemblies with optical and genetic mapping to construct chromosome-scale genomes for Japanese cypress (*Chamaecyparis obtusa*, 8.7 Gb), Japanese cedar (*Cryptomeria japonica*, 9.6 Gb), and Chinese fir (*Cunninghamia lanceolata*, 13.4 Gb). Additionally, we assembled and annotated their chloroplast and mitochondrial genomes. Comparative analysis of the nuclear genomes revealed that while synteny is largely conserved, distinct translocations and inversions occurred in chromosomes 2, 6, and 9. Notably, the significantly larger genome of *Cu. lanceolata* was associated with frequent tandem gene duplications rather than transposon expansion. These findings suggest that chromosomal rearrangements and segmental duplications played key roles in the divergence of these species. The genomic resources presented here including chromosome-scale sequences, gene annotations, and genetic maps will facilitate advanced conifer genetics and accelerate forest tree breeding programmes.

## 1. Introduction

The Spermatophyta, or seed plants, are broadly categorized into angiosperms and gymnosperms. Whereas angiosperms include more than 60 orders,^1^ gymnosperms currently consist of only 8 orders^2^: Araucariales, Cupressales, Cycadales, Ephedrales, Ginkgoales, Gnetales, Pinales, and Welwitschiales. Although the classification of both angiosperms and gymnosperms has been debated, molecular techniques have played a significant role in resolving these arguments. Gene sequences of organelle genomes, which are conserved across species yet slightly divergent, are recognized as DNA barcodes for identifying plant species.^3^ More recently, with great advancements in genome sequencing technologies, nuclear genome sequences at the chromosome level have been applied to the classification of angiosperms.^4^ Even though the genome sizes of angiosperms range from 100 Mb to 100 Gb,^6^ sequenced plant species have been biased towards those with small genome sizes (<1 Gb).^5^ In gymnosperms, on the other hand, most species possess massive genomes, approximately 10 Gb or more.^6^ Therefore, the assembly of chromosome-scale genome sequences in gymnosperms has lagged behind that of angiosperms.

Among gymnosperms, the Cupressaceae species belonging to the order Cupressales are vital resources for the forestry industry and for housing materials. In Japan, approximately 60% of the land is forested; of this, 40% comprises plantation (artificial) forests dominated by Japanese cedar (*Cryptomeria japonica*), followed by Japanese cypress (*Chamaecyparis obtusa*).^7,8^ Recently, Chinese fir (*Cunninghamia lanceolata*) has been recognized as a promising new woody resource due to its fast growth and desirable wood properties.^9^ However, breeding programmes for the Cupressaceae species are generally time- and labour-intensive because their generation times and harvest rotations often exceed 50 years. Genomic information would help accelerate these breeding programmes by identifying genes that control traits of importance to the forestry industry. In our previous study,^10^ we released draft genome sequences for *Ch. obtusa*, *Cr. japonica*, and *Cu. lanceolata*; however, these sequences were highly fragmented and lacked gene annotations.

Comparative analysis of genome structures using chromosome-scale assemblies can elucidate the evolution and speciation processes of species.^11^ Moreover, gene annotations, combined with chromosome-scale sequences, are valuable resources for identifying genes for use in breeding programmes.^5^ In this study, we extended the contiguity of the draft sequences for *Ch. obtusa*, *Cr. japonica*, and *Cu. lanceolata* using optical mapping and anchored the sequences to chromosomes using a genetic mapping strategy. Comparative genomics using the resulting chromosome-scale sequences provided insights into the evolution and speciation of the Cupressaceae species.

## 2. Materials and methods

### 2.1. Plant materials

For genome assemblies, we used the same individual trees of *Ch. obtusa* (tree ID: GFB00119), *Cr. japonica* (GFA01029), and *Cu. lanceolata* (GFHN00090) that were employed for the draft genome assemblies in our previous study.^10^ For genetic mapping, we utilized 4 mapping populations for *Ch. obtusa*, 2 for *Cr. japonica*, and 2 for *Cu. lanceolata*. The details of the mapping populations were listed in Table S1. For transcriptome analysis, 3 *Ch. obtusa* (GFB00064, GFB00092, and GFB00067), 1 *Cr. japonica* (GFA01185), and 1 *Cu. lanceolata* (GFHN00090) trees were used.

### 2.2. Optical mapping

Genomic DNA was extracted from young leaves of the 3 species using the Plant DNA Isolation Kit (Bionano Genomics, San Diego, California, United States), following the Bionano Prep Plant Tissue DNA Isolation Base Protocol. The isolated genomic DNA was treated with the DLE-1 enzyme and labelled with a fluorescent dye supplied in the DLS DNA Labeling Kit (Bionano Genomics). The labelled DNA was analyzed on the Saphyr Optical Genome Mapping Instrument (Bionano Genomics). The resulting molecules were assembled and merged with the draft assembly to generate hybrid scaffold sequences using Bionano Solve (version 3.7) (Bionano Genomics) with default parameters.

### 2.3. Genetic mapping and pseudomolecule sequence construction

For genetic mapping of *Ch. obtusa* and *Cu. lanceolata*, DNA was extracted from all individuals of the mapping populations and their parental lines using the sbeadex DNA extraction kit on the oKtopure system (LGC, United Kingdom). The extracted DNA was digested with PstI and MspI restriction endonucleases and subjected to ddRAD-Seq library preparation.^12^ The resulting libraries were sequenced on a DNBSEQ G400 instrument (MGI Tech) to generate 100-bp paired-end reads. Alternatively, specific populations were genotyped using GRAS-Di,^13^ and the sequence reads were processed as described below. After removing adapter sequences (AGATCGGAAGAGC for ddRAD-Seq or CTGTCTCTTATACACATCT for GRAS-Di) using fastx_clipper in the FASTX-Toolkit (version 0.0.14) and trimming low-quality reads (quality score <10) using PRINSEQ (version 0.20.4),^14^ high-quality reads were aligned to the hybrid scaffold sequences using Bowtie2 (version 2.3.5.1).^15^ High-confidence biallelic SNPs were identified using the mpileup and call options of BCFtools (version 1.9)^16^ and filtered using VCFtools (version 0.1.16)^17^ based on the following criteria: read depth ≥5; SNP quality = 999; minor allele frequency ≥0.2; and proportion of missing data <20%. Linkage analysis of the SNPs was performed using Lep-Map3 (version 0.2)^18^ to construct a genetic map. For genetic mapping of *Cr. japonica*, on the other hand, SNP genotyping analysis was performed using the SNP arrays, ie Axiom_Cj70K_ver. 1 and Axiom_Cj70K_ver. 2, on the Axiom genotyping system (Thermo Fisher Scientific, Waltham, Massachusetts, United States).^19^ Genetic maps for each parental meiosis were constructed with a double pseudo testcross strategy using the CP model implemented in JoinMap 4.1.^20^ In addition, we employed the *Cr. japonica* genetic map reported in our previous study.^19^ Finally, the hybrid scaffold sequences were anchored to the genetic map to establish chromosome-level pseudomolecule sequences using ALLMAPS (version 0.7.3).^21^

### 2.4. Transcriptome analysis with long- and short-read sequencing

Total RNA was extracted from 4 tissues (male and female strobili, cambium, and leaves) of *Ch. obtusa* using the Maxwell RSC Plant RNA Kit (Promega, Madison, Wisconsin, United States). For *Cr. japonica*, total RNA was extracted from leaves, shoots, and cambium using the RNeasy Plant Mini Kit (Qiagen, Hilden, Germany). For *Cu. lanceolata*, total RNA was extracted from 3 tissues (shoots, stems, and leaves) using the Maxwell RSC Plant RNA Kit (Promega).

For the *Ch. obtusa* and *Cu. lanceolata* samples, full-length RNA libraries for long-read cDNA sequencing were prepared using the Iso-Seq Express 2.0 Kit or the Kinnex Full-Length RNA Kit, and sequenced on the Sequel II or Revio systems (PacBio, Menlo Park, California, United States). The resulting full-length cDNA sequences were processed using the Iso-Seq pipeline (PacBio) to generate nonredundant transcript sequences. For the *Cr. japonica* samples, sequencing and analysis were performed as described by Mishima et al.^22^ In brief, size-selected cDNA libraries were prepared and sequenced using the PacBio RS II Iso-Seq platform (PacBio). Full-length nonchimeric reads and polished consensus isoforms were generated using the RS_IsoSeq pipeline (PacBio), and redundant isoforms were collapsed using CD-HIT to obtain nonredundant transcript sequences.

RNA libraries for short-read cDNA sequencing were prepared using the TruSeq Stranded mRNA Sample Preparation Kit (Illumina) and sequenced on a DNBSEQ G400 instrument (MGI Tech, Shenzhen, China). Adapter sequences and low-quality bases were trimmed as described above.

### 2.5. Gene and repetitive sequence annotation

Protein-coding genes were predicted in 3 steps: (i) transcript-based prediction was performed using GeneMarkS-T (version 5.1)^23^ with full-length cDNA sequences generated from Iso-Seq or Kinnex; (ii) ab initio prediction was conducted using Helixer (version 0.3.1)^24^; and (iii) BRAKER2 (version 2.1.5)^25^ was used in conjunction with short-read RNA-Seq data to identify genes missed by the first 2 methods. Gene prediction completeness was evaluated using Benchmarking Universal Single-Copy Orthologs (BUSCO) (version 5.5.0) with default parameters^26^ and the gymnosperms_odb10 lineage dataset.^27^

Repetitive sequences in the genome assemblies were identified using RepeatMasker (version 4.2.2) (https://www.repeatmasker.org) with the default parameters using reference repeat sequences from Repbase and de novo repeat libraries constructed using RepeatModeler (version 2.0.7) (https://www.repeatmasker.org).

### 2.6. Comparative analysis of the genome structure

The genome sequences of *Ch. obtusa*, *Cr. japonica*, and *Cu. lanceolata* from this study and those of *Cr. japonica*,^28^  *Sequoiadendron giganteum*,^29^  *Pinus densiflora*,^30^ and *P. tabuliformis*^31^ were compared using D-Genies (version 1.5.0).^32^ Pairwise collinear blocks in pseudomolecule sequences were identified using MCScanX^33^ with a match score threshold (-k) of 120 and an *E*-value cut-off of 10^−50^. The results were visualized using the synteny browser SynVisio.^34^

### 2.7. Functional annotation and gene ontology enrichment analysis

To evaluate the functional associations of duplicated genes in the *Cu. lanceolata* genome, we performed gene ontology (GO) enrichment analysis. First, functional annotations were assigned to 56,923 genes within the 11 pseudochromosome sequences of the *Cu. lanceolata* genome using InterProscan (version 5.77-108.0)^35^ with ‘-goterms’ option. We then conducted GO enrichment analysis of biological processes for 6,246 paralogous genes identified by MCScanX using clusterProfiler (version 4.20.0).^36^ Ancestor GO terms were added using the buildGOmap() function. GO enrichment analysis was performed using the enricher() function with default parameters. Finally, the simplify() function was used to reduce redundancy and select representative terms. For multiple testing correction, *P*-values were adjusted using the Benjamini–Hochberg method. GO terms with adjusted *P*-value less than 0.05 were considered significantly enriched.

### 2.8. Organelle genome assembly and annotation

Chloroplast and mitochondrial genomes were assembled using HiFi reads obtained in our previous study^10^ via Oatk (version 1.0), with the syncmer coverage threshold (-c) set to 30.^37^ While Oatk successfully generated both chloroplast and mitochondrial sequences for *Ch. obtusa* and *Cu. lanceolata*, it did not automatically assemble the mitochondrial genome of *Cr. japonica*. Consequently, we extracted mitochondrial unitigs from the Oatk assembly graph—identified via HMM search—and manually constructed a contig using Bandage (version 0.9.0).^38^

Chloroplast genes (protein-coding, tRNA, and rRNA) were annotated using LiftOn (version 1.0.5)^39^ based on alignments to published reference genomes: *Ch. obtusa* (GenBank accession PV469661.1), *Cr. japonica* (NC_010548.1), and *Cu. lanceolata* (NC_021437.1). Mitochondrial genes were similarly annotated using LiftOn, utilizing references from *Platycladus orientalis* (OL703044.1–OL703045.1), *Thuja sutchuenensis* (ON603305.1–ON603308.1), *Taxus chinensis* (NC_069591.1), *Taxus wallichiana* (NC_072607.1), and *Taxus cuspidata* (MN593023.1). Additionally, mitochondrial genes were predicted using PMGA,^40^ a pipeline for plant mitochondrial genome annotation. All gene structures were manually curated and corrected, and final annotations were visualized using OGDRAW (version 1.3.1).^41^

## 3. Results

### 3.1. Genome assembly and gene annotation for *Ch. obtusa*

In a previous study,^10^ we established a draft genome assembly for *Ch. obtusa* containing primary contigs spanning 8.5 Gb (N50 = 1.2 Mb) and alternate contigs spanning 7.8 Gb (N50 = 496.6 kb). To extend sequence contiguity, the combined 16.3 Gb of primary and alternate sequences were scaffolded using 2,058.1 Gb of optical mapping data (7,538,216 molecules; N50 length = 289.7 kb). The resulting hybrid scaffold assembly comprised 848 sequences with a total length of 9.6 Gb and an N50 of 54.2 Mb.

Subsequently, 4 genetic maps of *Ch. obtusa* were constructed via linkage analysis of SNPs across 4 mapping populations. Each genetic map consisted of 11 linkage groups (Table S2), corresponding to the basic chromosome number of *Ch. obtusa*. Based on these genetic maps, 378 hybrid scaffold sequences were anchored to the 11 linkage groups, establishing chromosome-scale pseudomolecule sequences spanning a total of 8.7 Gb (Table 1). The remaining 470 sequences (totalling 899.6 Mb) were unplaced. The complete BUSCO scores for the total assemblies including the unplaced sequences were 96.0%, while that for the 11 pseudomolecule sequences was 95.6% (Table 2).

**Table 1.** Statistics of the chromosome-scale genome assemblies for *Ch. obtusa*, *Cr. japonica*, and *Cu. lanceolata.*

Chromosome	*Ch. obtusa*		*Cr. japonica*		*Cu. lanceolata*	
	Length (bp)	% of length	Length (bp)	% of length	Length (bp)	% of length
Chr. 1	753,361,174	7.8	808,184,979	7.0	1,071,765,491	6.3
Chr. 2	643,942,463	6.7	787,791,099	6.8	1,098,708,507	6.5
Chr. 3	1,044,836,393	10.8	1,101,135,464	9.5	1,959,509,874	11.6
Chr. 4	815,834,336	8.5	803,702,909	6.9	1,350,266,392	8.0
Chr. 5	926,357,518	9.6	1,043,635,872	9.0	1,594,571,851	9.4
Chr. 6	607,243,501	6.3	760,652,447	6.5	906,465,489	5.4
Chr. 7	938,369,984	9.7	921,502,449	7.9	1,534,292,120	9.1
Chr. 8	754,287,409	7.8	787,691,810	6.8	922,755,377	5.5
Chr. 9	651,761,957	6.8	828,523,594	7.1	734,130,669	4.3
Chr. 10	888,336,793	9.2	950,971,039	8.2	1,497,158,838	8.8
Chr. 11	722,417,066	7.5	797,504,455	6.9	753,462,524	4.5
**Subtotal**	**8,746,748,594**	**90**.**7**	**9,591,296,117**	**82**.**5**	**13,423,087,132**	**79**.**3**
Unplaced contigs	899,622,983	9.3	2,035,395,917	17.5	3,497,085,311	20.7
**Total**	**9,646,371,577**	**100**.**0**	**11,626,692,034**	**100**.**0**	**16,920,172,443**	**100.0**

**Table 2.** Completeness assessment of genomes and gene predictions for *Ch. obtusa*, *Cr. japonica*, and *Cu. lanceolata.*

	*Ch. Obtusa*					*Cr. japonica*					*Cu. lanceolata*				
BUSCOs^a^	C (%)	S (%)	D (%)	F (%)	M (%)	C (%)	S (%)	D (%)	F (%)	M (%)	C (%)	S (%)	D (%)	F (%)	M (%)
Total genome assemblies	96.0	82.3	13.7	1.4	2.6	96.5	76.2	20.3	0.8	2.7	97.2	63.6	33.6	1.0	1.8
Pseudomolecule sequences	95.6	86.0	9.6	1.4	3.0	95.6	86.5	9.1	0.7	3.7	95.3	80.1	15.2	1.1	3.6
Genes predicted in the total genome assemblies	90.1	77.5	12.6	0.7	9.2	90.5	76.0	14.5	0.9	8.6	93.8	59.6	34.2	0.6	5.6
Genes predicted in the pseudomolecule sequences	89.2	79.0	10.2	0.7	10.1	87.6	79.2	8.4	0.9	11.5	90.9	71.7	19.2	0.8	8.3

^a^BUSCOs are categorized in complete (C), which consisted of complete and single-copy (S) and complete and duplicated (D), fragmented (F), and missing (M).

For gene prediction, transcriptome sequences were generated using both long- and short-read sequencing. Full-length cDNA sequences generated from this study (DDBJ DRA accession numbers: DRR933100–DRR933123) were clustered into 456,139 sequences containing 283,181 isoforms. From these, 27,384 nonredundant sequences were selected to predict 19,938 gene models, achieving a complete BUSCO score of 82.6%. In parallel, 1,728 million RNA-Seq reads from 96 samples (DRR933124–DRR933219) were used to predict 41,266 gene models (complete BUSCO score: 61.1%). Furthermore, the ab initio method predicted 50,494 gene models (complete BUSCO score: 60.6%). Finally, we merged these 3 datasets to select a total of 54,034 nonredundant gene models (Table 3), of which 49,391 genes were located on the 11 pseudomolecule sequences. The complete BUSCO scores for the 54,034 and 49,391 genes were 90.1% and 89.2%, respectively (Table 2).

**Table 3.** Summary of predicted genes in the genomes of *Ch. obtusa*, *Cr. japonica*, and *Cu. lanceolata.*

Chromosome	*Ch. obtusa*		*Cr. japonica*		*Cu. lanceolata*	
	#Genes	% of genes	#Genes	% of genes	#Genes	% of genes
Chr. 1	4,303	8.0	3,325	6.7	4,651	6.4
Chr. 2	4,042	7.5	3,323	6.7	4,808	6.7
Chr. 3	5,928	11.0	5,037	10.1	7,902	10.9
Chr. 4	4,605	8.5	3,503	7.0	5,688	7.9
Chr. 5	5,047	9.3	4,421	8.9	6,207	8.6
Chr. 6	3,337	6.2	2,958	5.9	4,320	6.0
Chr. 7	5,661	10.5	4,192	8.4	6,612	9.2
Chr. 8	3,829	7.1	2,992	6.0	3,963	5.5
Chr. 9	3,922	7.3	3,241	6.5	3,152	4.4
Chr. 10	4,957	9.2	3,943	7.9	6,135	8.5
Chr. 11	3,760	7.0	3,117	6.2	3,485	4.8
**Subtotal**	**49,391**	**91**.**4**	**40,052**	**80**.**3**	**56,923**	**78**.**9**
Unplaced contigs	4,643	8.6	9,847	19.7	15,246	21.1
**Total**	**54,034**	**100**.**0**	**49,899**	**100**.**0**	**72,169**	**100.0**

### 3.2. Genome assembly and gene annotation for *Cr. japonica*

The draft genome assembly for *Cr. japonica* comprised primary contigs spanning 9.2 Gb (N50 = 8.3 Mb) and alternate contigs spanning 8.7 Gb (N50 = 2.1 Mb).^10^ A total of 17.9 Gb of sequences was scaffolded using 2,151.5 Gb of optical mapping data (7,318,858 molecules; N50 length = 288.9 kb). The resulting hybrid scaffold assembly consisted of 805 sequences with a total length of 11.6 Gb and an N50 of 37.9 Mb.

A total of 5 genetic maps for *Cr. japonica* were used: 4 maps constructed using the 2 mapping populations (Table S1 and S3) and 1 reported in our previous study.^19^ Each genetic map consisted of 11 linkage groups (Table S3), corresponding to the basic chromosome number of *Cr. japonica*. Based on these maps, 294 hybrid scaffold sequences were anchored to the 11 linkage groups to establish chromosome-scale pseudomolecules spanning 9.6 Gb (Table 1). The remaining 511 sequences (2.0 Gb total) were unplaced. The complete BUSCO score for the total assemblies including the unplaced sequences was 96.5%, while that for the 11 pseudomolecule sequences was 95.6% (Table 2). The genome structure of the *Cr. japonica* from this study was basically conserved with that published in the previous study^28^ (Figure S1), suggesting that the chromosome-scale assemblies in both studies would be accurate.

For gene prediction, we used 75,348 isoforms derived from full-length cDNA sequences generated from this study (DRR1057412–DRR1057439) to predict 14,449 gene models (BUSCO completeness: 83.4%). Next, we utilized 34,731 unigene sequences from our previous study^19^ to predict 25,856 gene models (BUSCO completeness: 59.9%). Additionally, the ab initio method predicted 58,119 gene models (BUSCO completeness: 61.4%). These 3 datasets were merged to select a final set of 49,899 nonredundant gene models (Table 3), of which 40,052 genes were located on the 11 pseudomolecule sequences. The complete BUSCO scores for the 49,899 and the 40,052 genes were 90.5% and 87.6%, respectively (Table 2).

### 3.3. Genome assembly and gene annotation for *Cu. lanceolata*

The draft genome assembly for *Cu. lanceolata* contained primary contigs spanning 11.5 Gb (N50 = 11.7 Mb) and alternate contigs spanning 11.0 Gb (N50 = 2.9 Mb).^10^ A total of 22.5 Gb of sequence was scaffolded using 2,191.6 Gb of optical mapping data (5,368,583 molecules; N50 = 397.9 kb). The resulting hybrid scaffold assembly comprised 822 sequences with a total length of 16.9 Gb and an N50 of 31.8 Mb.

Two genetic maps for *Cu. lanceolata* were constructed using 2 distinct mapping populations. One genetic map consisted of 11 linkage groups (Table S4), corresponding to the basic chromosome number of *Cu. lanceolata*. The second map consisted of 16 linkage groups (Table S4); in 6 cases, pairs of linkage groups were assigned to a single chromosome sequence, while 1 chromosome was not represented by any linkage group. Ultimately, 298 hybrid scaffold sequences were anchored to the 11 linkage groups to establish chromosome-scale pseudomolecule sequences spanning a total of 13.4 Gb (Table 1). The remaining 524 sequences (3.5 Gb total) were unplaced. The complete BUSCO score for the total assemblies including the unplaced sequences was 97.2%, while that for the 11 pseudomolecule sequences was 95.3% (Table 2).

For gene prediction, we utilized 1,088,667 nonredundant full-length cDNA sequences generated from this study (DRR933220–DRR933225) to predict 35,735 gene models (complete BUSCO score: 90.5%). Next, 113.2 million RNA-Seq reads from shoots, stems, and leaf samples were downloaded from a public database (NCBI SRA accession numbers SRR5282557–SRR5282559) and used to predict 47,922 gene models (complete BUSCO score: 62.0%). Additionally, the ab initio method predicted 45,934 gene models (complete BUSCO score: 40.6%). These 3 datasets were merged to select a total of 72,169 nonredundant gene models (Table 3), of which 56,923 genes were located on the 11 pseudomolecule sequences. The complete BUSCO scores for the 72,169 and the 56,923 genes were 93.8% and 90.9%, respectively (Table 2).

### 3.4. Repetitive sequence analysis

Repetitive sequences account for 77.5%, 80.5%, and 80.9% of the genomes of *Ch. obtusa*, *Cr. japonica*, and *Cu. lanceolata*, respectively. LTR retrotransposons were the most abundant class of repetitive sequences (48.5%–53.6%), followed by unclassified repeats (14.3%–21.6%), DNA transposons (5.2%–6.8%), and LINEs (3.3%–4.9%) (Table 4).

**Table 4.** Classification and abundance of repetitive sequences in the genomes of *Ch. obtusa*, *Cr. japonica*, and *Cu. lanceolata.*

Repeat type	*Ch. obtusa*			*Cr. japonica*			*Cu. lanceolata*		
	#Elements	Length (bp)	%	#Elements	Length (bp)	%	#Elements	Length (bp)	%
SINEs	11,430	1,313,246	0.0	12,828	2,413,789	0.0	50,707	15,072,777	0.1
LINEs	536,344	356,067,530	3.7	647,369	568,704,286	4.9	793,194	558,315,070	3.3
LTR elements	2,900,360	4,680,905,729	48.5	3,606,721	6,235,301,883	53.6	6,005,684	8,319,335,602	49.2
DNA transposons	738,498	505,285,736	5.2	1,137,352	785,356,255	6.8	1,277,378	903,211,596	5.3
Small RNA	9,934	4,679,246	0.1	23,970	9,465,882	0.1	68,538	20,640,162	0.1
Satellites	29,335	26,779,510	0.3	19,979	10,042,049	0.1	75,861	45,335,388	0.3
Simple repeats	764,656	55,374,637	0.6	902,078	50,675,895	0.4	980,278	58,458,100	0.4
Low complexity	125,181	8,185,083	0.1	144,624	8,419,661	0.1	166,010	10,190,191	0.1
Unclassified	3,210,805	1,807,553,850	18.7	3,289,375	1,666,957,226	14.3	8,058,531	3,655,997,225	21.6

### 3.5. Comparative analysis of the 3 coniferous genomes

**Fig. 1.**
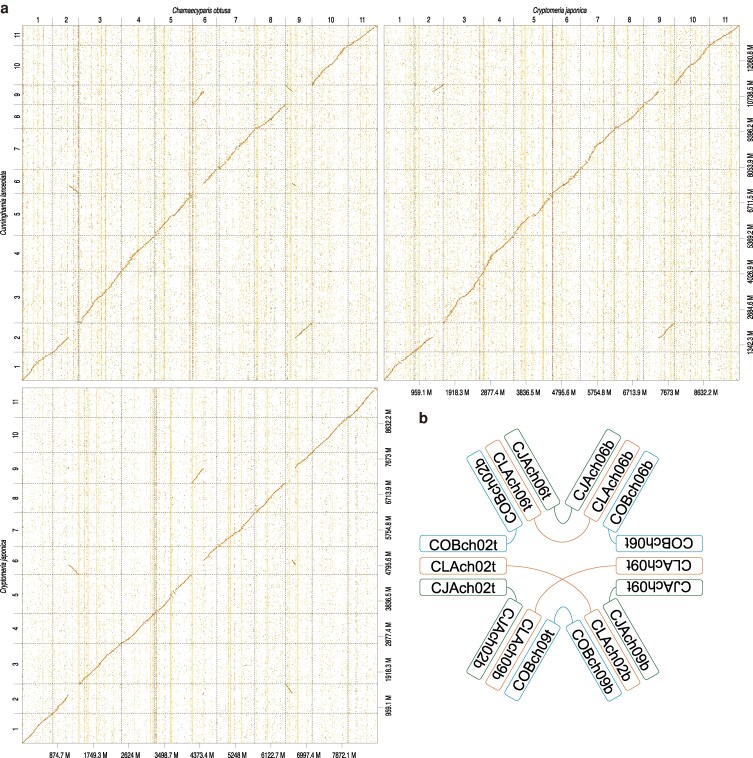
Comparative analysis of genome sequences and structures of *Ch. obtusa*, *Cr. japonica*, and *Cu. lanceolata*. a) Chromosome numbers are indicated on the top (*x* axis) and left (*y* axis). Genome sizes (Mb) are shown on the bottom (*x* axis) and right (*y* axis). b) Three chromosomes with significant rearrangements. Suffixes t and b indicate top (5' end) and bottom (3' end) of the chromosome sequences, respectively.

The chromosome-scale genome sequences of the 3 species were compared pairwise (Fig. 1a). This comparison revealed that while the structures of 8 chromosomes (Chr 1, 3, 4, 5, 7, 8, 10, and 11) were well conserved across all 3 species, 3 chromosomes (Chr 2, 6, and 9) underwent significant rearrangements (Fig. 1b). Because the genome sequences have not been associated with karyotypes, we tentatively designate the 5′- and 3′-ends of the sequences as top and bottom, respectively, hereafter. In *Ch. obtusa* chromosome 2, the top region (COBch02t) was conserved across all 3 species. However, the bottom region (COBch02b) was inverted and translocated to the top of chromosome 6 in both *Cr. japonica* (CJAch06t) and *Cu. lanceolata* (CLAch06t). Concurrently, regarding *Ch. obtusa* chromosome 6, the bottom region was conserved across species, while the top region (COBch06t) was translocated to the top of chromosome 9 in *Cr. japonica* (CJAch09t) and *Cu. lanceolata* (CLAch09t). The structure of *Ch. obtusa* chromosome 9 was more complex. The top region (COBch09t) was inverted and translocated to the bottom of *Cr. japonica* chromosome 2 (CJAch02b) and the bottom of *Cu. lanceolata* chromosome 9 (CLAch09b). Conversely, the bottom region (COBch09b) was translocated to the bottom of *Cr. japonica* chromosome 9 (CJAch09b) and the bottom of *Cu. lanceolata* chromosome 2 (CLAch02b). These patterns indicate a reciprocal translocation event involving the bottom regions of chromosomes 2 and 9 between *Cr. japonica* and *Cu. lanceolata*. Subsequently, the genome sequences of the 3 species were compared with *S. giganteum*, also a member of the Cupressaceae, to find an additional translocation between chromosomes 3 and 7 (Figure S2). We also compared the three Cupressaceae genomes with the chromosome-scale assemblies of *P. densiflora* and *P. tabuliformis*, representing Pinaceae. In contrast to the comparisons within Cupressaceae, these cross-family comparisons did not reveal robust chromosome-scale collinearity under the present analytical conditions.

**Fig. 2.**
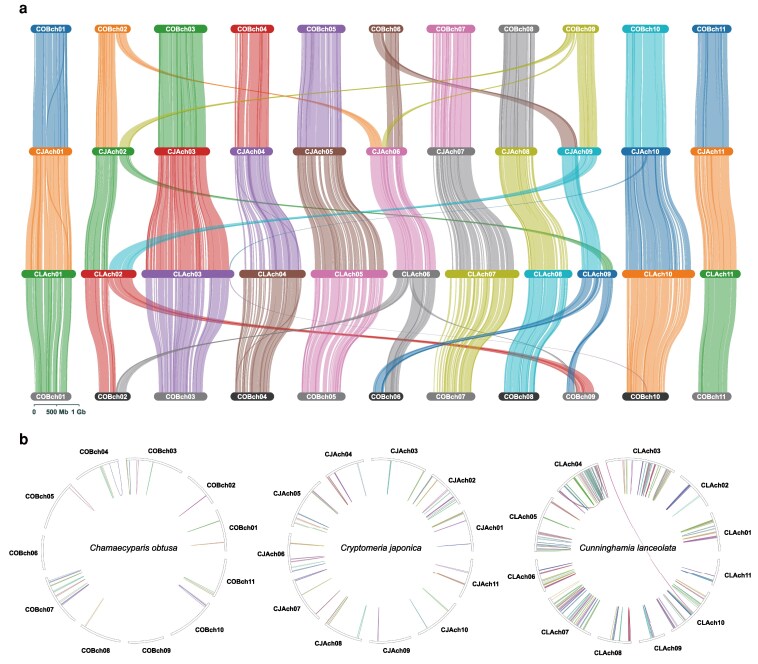
Synteny analysis of *Ch. obtusa*, *Cr. japonica*, and *Cu. lanceolata*. a) Lines connect pairwise collinear blocks between the genomes of *Ch. obtusa*, *Cr. japonica*, and *Cu. lanceolata*. b) Lines connect collinear blocks within each genome to visualize tandem duplications.

Synteny analysis (Fig. 2a) identified 216 pairwise collinear blocks (17,449 gene pairs) between *Ch. obtusa* and *Cr. japonica*. Similarly, 271 blocks (16,918 gene pairs) were identified between *Cr. japonica* and *Cu. lanceolata*, and 254 blocks (16,022 gene pairs) were identified between *Cu. lanceolata* and *Ch. obtusa*. Finally, the analysis detected tandem gene duplications (Fig. 2b), revealing 23 blocks (525 gene pairs) in *Ch. obtusa*, 37 blocks (924 gene pairs) in *Cr. japonica*, and a notably higher frequency of 89 blocks (3,149 gene pairs) in *Cu. lanceolata*.

### 3.6. GO enrichment analysis for duplicated genes in the *Cu. lanceolata* genome

Among the 56,923 genes within the 11 pseudomolecule sequences of the *Cu. lanceolata* genome, 35,094 genes were assigned at least 1 GO ID in the functional annotation. As a result of GO enrichment analysis for 6,246 paralogous genes in the *Cu. lanceolata* genome, 49 GO terms were significantly enriched, including response to oxidative stress, phosphate ion transport, and cellulose biosynthetic process (Figure S3 and Table S5).

### 3.7. Organelle genome assemblies and gene annotations

The chloroplast genomes of *Ch. obtusa*, *Cr. japonica*, and *Cu. lanceolata* were assembled as single circular sequences with lengths of 127,726 bp, 131,741 bp, and 135,331 bp, respectively (Figure S4). All 3 chloroplast genome sequences lacked 1 copy of the inverted repeats, which are found in the chloroplast genomes of most plants, including not only angiosperms but also gymnosperms, such as *Cycas* and *Ginkgo*, but are reduced or lost in the Pinaceae and the Cupressaceae.^42,43^ The *Ch. obtusa* chloroplast genome contained 120 genes (83 protein-coding, 33 tRNA, and 4 rRNA). In *Cr. japonica*, 118 genes were annotated (82 protein-coding, 32 tRNA, and 4 rRNA), while in *Cu. lanceolata*, 120 genes were annotated (81 protein-coding, 35 tRNA, and 4 rRNA).

The mitochondrial genomes of 3 species were large and exhibited complex assembly graphs with multiple nodes and connections (Figures S5 and S6). The mitochondrial genome of *Ch. obtusa* was assembled as a single linear sequence of 1,908,789 bp, and that of *Cr. japonica* as a single linear sequence of 3,743,212 bp (Figure S5). In contrast, the mitochondrial genome of *Cu. lanceolata* consisted of 6 sequences with a total length of 1,993,458 bp, comprising 1 circular sequence and 5 linear sequences (Figure S5). Gene annotation identified 70 genes in the *Ch. obtusa* mitochondrial genome (33 protein-coding, 26 tRNA, 11 rRNA); 71 genes in *Cr. japonica* (36 protein-coding, 22 tRNA, 13 rRNA); and 65 genes in *Cu. lanceolata* (36 protein-coding, 23 tRNA, 6 rRNA).

## 4. Discussion

We present chromosome-scale genome assemblies for 3 species of the Cupressaceae: *Ch. obtusa*, *Cr. japonica*, and *Cu. lanceolata* (Table 1). Consistent with previous genome size estimates,^10^ the resulting assembly length of *Cu. lanceolata* (13.4 Gb) was significantly larger than those of *Ch. obtusa* (8.7 Gb) and *Cr. japonica* (9.6 Gb). Since the proportion of repetitive sequences remained approximately 80% across all 3 genomes (Table 4), the larger genome size of *Cu. lanceolata* appears to be driven by the multiplication of genomic segments containing both repetitive and non-repetitive sequences, rather than solely by a burst of transposon activity. This hypothesis is supported by the higher number of predicted genes in the pseudomolecule sequences of *Cu. lanceolata* (56,923) compared to *Ch. obtusa* (49,391) and *Cr. japonica* (40,052) (Table 3), as well as its elevated duplicated BUSCO score (19.2%) relative to *Ch. obtusa* (10.2%) and *Cr. japonica* (8.4%) (Table 2). These results, in addition to the consistency between the pseudomolecule sequence length and the previous genome size estimates,^10^ suggest that frequent tandem gene duplications (Fig. 2b) have contributed to the expansion of both genome size and gene content in *Cu. lanceolata*. Notably, unplaced contigs (assembled sequences not integrated into pseudomolecules) also contained genes (Table 3). This likely contributed to the elevated complete and duplicated BUSCO scores (Table 2), suggesting that these unplaced sequences contained allelic regions of the alternative haplotype in the diploid genome.

Comparative genome analysis revealed extensive macro-collinearity among the analyzed Cupressaceae species, with specific structural rearrangements providing new insights into conifer evolution. Consistent with previous findings,^44^ rearrangements were observed in 3 chromosomes (2, 6, and 9) between *Ch. obtusa* and *Cr. japonica*, as well as between *Ch. obtusa* and *Cu. lanceolata*. In contrast, rearrangements were restricted to 2 chromosomes (2 and 9) between *Cr. japonica* and *Cu. lanceolata* (Fig. 1). While one might infer a closer phylogenetic relationship between *Cr. japonica* and *Cu. lanceolata*, molecular phylogenetic studies^45^ indicate that *Cunninghamia* diverged first during the Triassic period (∼243.7 Mya), followed by the divergence of *Chamaecyparis* and *Cryptomeria* during the Jurassic period (∼171.3 Mya). This timeline suggests that the reciprocal translocations involving chromosomes 2 and 9 occurred during or after the Triassic, whereas the rearrangements in chromosomes 2, 6, and 9 arose during or after the Jurassic. Furthermore, the *Cu. lanceolata* genome was largely collinear with *S. giganteum*, except for a translocation between chromosomes 3 and 7 (Figure S2). This suggests that the chromosome 3 to 7 translocation occurred specifically in the *S. giganteum* lineage after its divergence from the common ancestor shared with *Cu. lanceolata*, likely during or after the Jurassic period (∼192.2 Mya). Along with these macro-structural changes, local tandem duplications in *Cu. lanceolata* were enriched for genes associated with oxidative stress responses, phosphate ion transport, and cellulose biosynthesis (Fig. 2b), suggesting that local duplication contributed to environmental adaptation and wood formation. Together, these chromosomal rearrangements and tandem gene duplications (Figs. 1 and 2) highlight the complex evolutionary processes in conifers, underscoring the need for additional chromosome-scale genomes from diverse taxa to clarify lineage-specific evolutionary patterns in gymnosperms.

In addition to the nuclear genome, assembly of the mitochondrial genome in gymnosperm species is challenging due to their large genome size and structural complexity.^46,47^ The assembly graphs of the mitochondrial genomes for *Ch. obtusa*, *Cr. japonica*, and *Cu. lanceolata* consisted of many nodes with multiple connections (Figure S6). Plant mitochondrial DNA molecules can take various structures, including circular, linear, and branched forms.^48,49^ Structural variations can be generated through recombination mediated by repeat sequences in mitochondrial genomes.^50^ The complex assembly graphs likely reflect the presence of structural variations in the mitochondrial genomes of *Ch. obtusa*, *Cr. japonica*, and *Cu. lanceolata* (Figure S6). Note that the assembled mitochondrial genomes in this study represent one of several possible conformations and do not necessarily reflect the actual conformation of mitochondrial DNA molecules in the mitochondria.

Despite the large size (Table 1) and high proportion of repetitive sequences (∼80%), including transposons (Table 4), in these nuclear genomes, the comparative analysis identified few chromosomal rearrangements as reported.^51^ Although transposable elements can drive chromosomal rearrangements,^52,53^ only 3 breakpoints on chromosomes 2, 6, and 9 were identified across these 3 Cupressaceae species (Fig. 1). Even with the inclusion of *S. giganteum* in the subsequent analysis, only 1 additional breakpoint was detected between chromosomes 3 and 7 (Figure S2). This genomic stability contrasts with the highly complex mitochondrial genomes observed in these species (Figure S4). Furthermore, genomic rearrangements are frequently reported in angiosperms at the family level, such as within the Fabaceae^54^ and Solanaceae,^55^ despite their shorter divergence times (<100 Mya). Therefore, we propose that comparative genomic structure analysis of such macro-collinearity could offer an alternative framework for elucidating the evolutionary history of both angiosperms and gymnosperms.^5,56^ Further genome sequencing of diverse gymnosperm species will be essential to resolve the current paradoxes surrounding genome size, divergence time, and nuclear–organelle genome complexity in gymnosperms.

The genome resources presented here will provide useful information for extending previous genetic and marker-based studies of Cupressaceae forest trees to genome-wide analyses. Previous population genetic studies have revealed genetic structure, regional genetic diversity, and candidate loci associated with local adaptation in natural populations of *Cr. japonica* and *Ch. obtusa*, demonstrating the importance of molecular markers for the management and conservation of these species.^8,57,58^ In *Ch. obtusa*, genetic analyses of natural populations and plus-tree groups, microsatellite-based studies of fragmented natural populations and recent linkage-map development have provided a basis for marker-based management and breeding applications.^44,59^ In *Cr. japonica*, genome-wide studies using unrelated first-generation plus trees have shown that dense SNP markers can be applied to GWAS and genomic prediction for traits such as growth, wood properties, and male fecundity.^60^ Chromosome-level genome assembly and marker development for male sterility in *Cr. japonica* have also provided useful resources for connecting markers with candidate genes and breeding targets.^19,28^ For *Cu. lanceolata*, previous SSR- and RAD-seq-based studies have shown that molecular markers are useful for assessing genetic diversity and population structure in breeding populations and clonal resources.^61–63^ Thus, the chromosome-scale assemblies and gene annotations obtained in this study, together with these existing resources, will be useful for evaluating genetic diversity, population structure, and regional differentiation in natural populations, breeding materials, seed orchards, and plantations of Cupressaceae conifers.

In conclusion, this study provides essential genomic resources, including chromosome-scale assemblies, gene annotations, and genetic maps, for *Ch. obtusa*, *Cr. japonica*, and *Cu. lanceolata*. The availability of multiple genome assemblies, facilitating pan-genome analysis, is crucial for understanding the genetic mechanisms underlying species diversity.^11^ The resources presented here, combined with existing data, will facilitate further research into the evolution and speciation of these conifers and serve as a crucial milestone to accelerate the molecular breeding and genetic improvement of these economically important trees.

## Supplementary Material

dsag010_Supplementary_Data

## Data Availability

Raw sequence reads and the assembled sequences were deposited in DDBJ (BioProject accession number PRJDB40558). The genome assembly files and gene annotation files for *Ch. obtusa* (COB_r1.0.genome), *Cr. japonica* (CJA_r1.0.genome), and *Cu. lanceolata* (CLA_r1.0.genome) are available in Kazusa Genome Atlas (https://genome.kazusa.or.jp).

## References

[1] The Angiosperm Phylogeny Group et al An update of the Angiosperm Phylogeny Group classification for the orders and families of flowering plants: APG IV. Bot J Linn Soc. 2016:181:1–20. 10.1111/boj.12385

[2] Christenhusz MJM et al A new classification and linear sequence of extant gymnosperms. Phytotaxa. 2011:19:55–70. 10.11646/phytotaxa.19.1.3

[3] CBOL Plant Working Group . A DNA barcode for land plants. Proc Natl Acad Sci U S A. 2009:106:12794–12797. 10.1073/pnas.0905845106 19666622 PMC2722355

[4] Zhao T et al Whole-genome microsynteny-based phylogeny of angiosperms. Nat Commun. 2021:12:3498. 10.1038/s41467-021-23665-0 34108452 PMC8190143

[5] Shirasawa K, Harada D, Hirakawa H, Isobe S, Kole C. Chromosome-level *de novo* genome assemblies of over 100 plant species. Breed Sci. 2021:71:117–124. 10.1270/jsbbs.20146 34377059 PMC8329882

[6] Pellicer J, Leitch IJ. The plant DNA C-values database (release 7.1): an updated online repository of plant genome size data for comparative studies. New Phytol. 2020:226:301–305. 10.1111/nph.16261 31608445

[7] Forestry Agency . Annual report on forest and forestry in Japan. Ministry of Agriculture, Forestry and Fisheries; 2022.

[8] Tsumura Y et al Genetic diversity and the genetic structure of natural populations of Chamaecyparis obtusa: implications for management and conservation. Heredity (Edinb). 2007:99:161–172. 10.1038/sj.hdy.6800978 17473864

[9] Fujisawa Y . The future of forestry and Chinese fir. For Genet Tree Breed. 2017:6:132–136. 10.32135/fgtb.6.4_132

[10] Shirasawa K et al Haplotype-resolved *de novo* genome assemblies of four coniferous tree species. J Forest Res. 2024:29:151–157. 10.1080/13416979.2023.2267304

[11] Aoyagi Blue Y, Iimura H, Sato MP, Shirasawa K. The impact of telomere-to-telomere genome assembly in the plant pan-genomics era. Breed Sci. 2025:75:3–12. 10.1270/jsbbs.24065 40585571 PMC12203254

[12] Shirasawa K, Hirakawa H, Isobe S. Analytical workflow of double-digest restriction site-associated DNA sequencing based on empirical and in silico optimization in tomato. DNA Res. 2016:23:145–153. 10.1093/dnares/dsw004 26932983 PMC4833422

[13] Enoki, H., and Takeuchi, Y. New genotyping technology, GRAS-Di, using next generation sequencer. In: Plant & Animal Genome XXVI Poster Abstracts; 2018. p. P0153.

[14] Schmieder R, Edwards R. Quality control and preprocessing of metagenomic datasets. Bioinformatics. 2011:27:863–864. 10.1093/bioinformatics/btr026 21278185 PMC3051327

[15] Langmead B, Salzberg SL. Fast gapped-read alignment with Bowtie 2. Nat Methods. 2012:9:357–359. 10.1038/nmeth.1923 22388286 PMC3322381

[16] Li H . A statistical framework for SNP calling, mutation discovery, association mapping and population genetical parameter estimation from sequencing data. Bioinformatics. 2011:27:2987–2993. 10.1093/bioinformatics/btr509 21903627 PMC3198575

[17] Danecek P et al The variant call format and VCFtools. Bioinformatics. 2011:27:2156–2158. 10.1093/bioinformatics/btr330 21653522 PMC3137218

[18] Rastas P . Lep-MAP3: robust linkage mapping even for low-coverage whole genome sequencing data. Bioinformatics. 2017:33:3726–3732. 10.1093/bioinformatics/btx494 29036272

[19] Mishima K et al Identification of novel putative causative genes and genetic marker for male sterility in Japanese cedar (Cryptomeria japonica D.Don). BMC Genomics. 2018:19:277. 10.1186/s12864-018-4581-5 29685102 PMC5914023

[20] Van Ooijen JW . JoinMAP 4, software for the calculation of genetic linkage maps in experimental populations. Kyazma B.V.; 2006.

[21] Tang H et al ALLMAPS: robust scaffold ordering based on multiple maps. Genome Biol. 2015:16:3. 10.1186/s13059-014-0573-1 25583564 PMC4305236

[22] Mishima K et al Comprehensive collection of genes and comparative analysis of full-length transcriptome sequences from Japanese larch (Larix kaempferi) and Kuril larch (Larix gmelinii var. japonica). BMC Plant Biol. 2022:22:470. 10.1186/s12870-022-03862-9 36192701 PMC9531402

[23] Besemer J, Borodovsky M. GeneMark: web software for gene finding in prokaryotes, eukaryotes and viruses. Nucleic Acids Res. 2005:33:W451–W454. 10.1093/nar/gki487 15980510 PMC1160247

[24] Stiehler F et al Helixer: cross-species gene annotation of large eukaryotic genomes using deep learning. Bioinformatics. 2021:36:5291–5298. 10.1093/bioinformatics/btaa1044 33325516 PMC8016489

[25] Brůna T, Hoff KJ, Lomsadze A, Stanke M, Borodovsky M. BRAKER2: automatic eukaryotic genome annotation with GeneMark-EP+ and AUGUSTUS supported by a protein database. NAR Genom Bioinform. 2021:3:lqaa108. 10.1093/nargab/lqaa108 33575650 PMC7787252

[26] Simão FA, Waterhouse RM, Ioannidis P, Kriventseva EV, Zdobnov EM. BUSCO: assessing genome assembly and annotation completeness with single-copy orthologs. Bioinformatics. 2015:31:3210–3212. 10.1093/bioinformatics/btv351 26059717

[27] Wu J-J, Han Y-W, Lin C-F, Cai J, Zhao Y-P. Benchmarking gene set of gymnosperms for assessing genome and annotation completeness in BUSCO. Hortic Res. 2023:10:uhad165. 10.1093/hr/uhad165 37731863 PMC10508034

[28] Fujino T et al A chromosome-level genome assembly of a model conifer plant, the Japanese cedar, Cryptomeria japonica D. Don. BMC Genomics. 2024:25:1039. 10.1186/s12864-024-10929-4 39501145 PMC11539532

[29] Scott AD et al A reference genome sequence for giant sequoia. G3 (Bethesda). 2020:10:3907–3919. 10.1534/g3.120.401612 32948606 PMC7642918

[30] Jang M-J et al Haplotype-resolved genome assembly and resequencing analysis provide insights into genome evolution and allelic imbalance in Pinus densiflora. Nat Genet. 2024:56:2551–2561. 10.1038/s41588-024-01944-y 39428511

[31] Niu S et al The Chinese pine genome and methylome unveil key features of conifer evolution. Cell. 2022:185:204–217.e14. 10.1016/j.cell.2021.12.006 34965378

[32] Cabanettes F, Klopp C. D-GENIES: dot plot large genomes in an interactive, efficient and simple way. PeerJ. 2018:6:e4958. 10.7717/peerj.4958 29888139 PMC5991294

[33] Wang Y et al MCScanX: a toolkit for detection and evolutionary analysis of gene synteny and collinearity. Nucleic Acids Res. 2012:40:e49. 10.1093/nar/gkr1293 22217600 PMC3326336

[34] Bandi V, Gutwin C. Interactive exploration of genomic conservation. In: Proc. Graph. Interface; 2020. p. 74–83.

[35] Jones P et al InterProScan 5: genome-scale protein function classification. Bioinformatics. 2014:30:1236–1240. 10.1093/bioinformatics/btu031 24451626 PMC3998142

[36] Wu T et al clusterProfiler 4.0: a universal enrichment tool for interpreting omics data. Innovation (Camb). 2021:2:100141. 10.1016/j.xinn.2021.100141 34557778 PMC8454663

[37] Zhou C, Brown M, Blaxter M, McCarthy SA, Durbin R. Oatk: a de novo assembly tool for complex plant organelle genomes. Genome Biol. 2025:26:235. 10.1186/s13059-025-03676-6 40775726 PMC12329965

[38] Wick RR, Schultz MB, Zobel J, Holt KE. Bandage: interactive visualization of de novo genome assemblies. Bioinformatics. 2015:31:3350–3352. 10.1093/bioinformatics/btv383 26099265 PMC4595904

[39] Chao K-H et al Combining DNA and protein alignments to improve genome annotation with LiftOn. Genome Res. 2025:35:311–325. 10.1101/gr.279620.124 39730188 PMC11874971

[40] Li J, Ni Y, Lu Q, Chen H, Liu C. PMGA: a plant mitochondrial genome annotator. Plant Commun. 2025:6:101191. 10.1016/j.xplc.2024.101191 39521957 PMC11956084

[41] Greiner S, Lehwark P, Bock R. OrganellarGenomeDRAW (OGDRAW) version 1.3.1: expanded toolkit for the graphical visualization of organellar genomes. Nucleic Acids Res. 2019:47:W59–W64. 10.1093/nar/gkz238 30949694 PMC6602502

[42] Hirao T, Watanabe A, Kurita M, Kondo T, Takata K. Complete nucleotide sequence of the Cryptomeria japonica D. Don. chloroplast genome and comparative chloroplast genomics: diversified genomic structure of coniferous species. BMC Plant Biol. 2008:8:70. 10.1186/1471-2229-8-70 18570682 PMC2443145

[43] Strauss SH, Palmer JD, Howe GT, Doerksen AH. Chloroplast genomes of two conifers lack a large inverted repeat and are extensively rearranged. Proc Natl Acad Sci U S A. 1988:85:3898–3902. 10.1073/pnas.85.11.3898 2836862 PMC280327

[44] Dogan G et al New linkage maps for the Cupressaceae species *Chamaecyparis obtusa* and *Thujopsis dolabrata* var. *hondae* and comparison with the linkage map of *Cryptomeria japonica*. J Forest Res. 2024:29:279–287. 10.1080/13416979.2024.2314829

[45] Liu X-Q, Xia X-M, Chen L, Wang X-Q. Phylogeny and evolution of Cupressaceae: updates on intergeneric relationships and new insights on ancient intergeneric hybridization. Mol Phylogenet Evol. 2022:177:107606. 10.1016/j.ympev.2022.107606 35952837

[46] Jackman SD et al Complete mitochondrial genome of a gymnosperm, Sitka spruce (Picea sitchensis), indicates a complex physical structure. Genome Biol Evol. 2020:12:1174–1179. 10.1093/gbe/evaa108 32449750 PMC7486957

[47] Huang K et al Super-large record-breaking mitochondrial genome of Cathaya argyrophylla in Pinaceae. Front Plant Sci. 2025:16:1556332. 10.3389/fpls.2025.1556332 40612618 PMC12222119

[48] Manchekar M et al DNA recombination activity in soybean mitochondria. J Mol Biol. 2006:356:288–299. 10.1016/j.jmb.2005.11.070 16376379

[49] Kozik A et al The alternative reality of plant mitochondrial DNA: one ring does not rule them all. PLoS Genet. 2019:15:e1008373. 10.1371/journal.pgen.1008373 31469821 PMC6742443

[50] Gualberto JM, Newton KJ. Plant mitochondrial genomes: dynamics and mechanisms of mutation. Annu Rev Plant Biol. 2017:68:225–252. 10.1146/annurev-arplant-043015-112232 28226235

[51] Zhang R-G et al Convergent patterns of karyotype evolution underlying karyotype uniformity in conifers. Adv Sci (Weinh). 2025:12:e2411098. 10.1002/advs.202411098 39721021 PMC11831501

[52] Walker EL, Robbins TP, Bureau TE, Kermicle J, Dellaporta SL. Transposon-mediated chromosomal rearrangements and gene duplications in the formation of the maize R-r complex. EMBO J. 1995:14:2350–2363. 10.1002/j.1460-2075.1995.tb07230.x 7774593 PMC398344

[53] Balachandran P et al Transposable element-mediated rearrangements are prevalent in human genomes. Nat Commun. 2022:13:7115. 10.1038/s41467-022-34810-8 36402840 PMC9675761

[54] Wang L et al Pangenome analysis provides insights into legume evolution and breeding. Nat Genet. 2025:57:2052–2061. 10.1038/s41588-025-02280-5 40738999

[55] Zhang L et al Solanaceae pan-genomes reveal extensive fractionation and functional innovation of duplicated genes. Plant Commun. 2025:6:101231. 10.1016/j.xplc.2024.101231 39719828 PMC11956106

[56] Shirasawa K et al Chromosome-level genome assembly of Japanese chestnut (Castanea crenata Sieb. et Zucc.) reveals conserved chromosomal segments in woody rosids. DNA Res. 2021:28:dsab016. 10.1093/dnares/dsab016 34424280 PMC8435554

[57] Tsumura Y et al Genome scan to detect genetic structure and adaptive genes of natural populations of Cryptomeria japonica. Genetics. 2007:176:2393–2403. 10.1534/genetics.107.072652 17565947 PMC1950640

[58] Tsumura Y, Uchiyama K, Moriguchi Y, Ueno S, Ihara-Ujino T. Genome scanning for detecting adaptive genes along environmental gradients in the Japanese conifer, Cryptomeria japonica. Heredity (Edinb). 2012:109:349–360. 10.1038/hdy.2012.50 22929151 PMC3499843

[59] Matsumoto A, Uchida K, Taguchi Y, Tani N, Tsumura Y. Genetic diversity and structure of natural fragmented Chamaecyparis obtusa populations as revealed by microsatellite markers. J Plant Res. 2010:123:689–699. 10.1007/s10265-009-0299-4 20091205

[60] Hiraoka Y et al Potential of genome-wide studies in unrelated plus trees of a coniferous Species, *Cryptomeria japonica* (Japanese Cedar). Front Plant Sci. 2018:9:1322. 10.3389/fpls.2018.01322 30254658 PMC6141754

[61] Lin E et al Genome survey of Chinese fir (Cunninghamia lanceolata): identification of genomic SSRs and demonstration of their utility in genetic diversity analysis. Sci Rep. 2020:10:4698. 10.1038/s41598-020-61611-0 32170167 PMC7070021

[62] Jing Y et al Genetic diversity and structure of the 4th cycle breeding population of Chinese fir (*Cunninghamia lanceolata* (lamb.) hook). Front Plant Sci. 2023:14:1106615. 10.3389/fpls.2023.1106615 36778690 PMC9911867

[63] Zhao B et al Development of an advanced-generation multi-objective breeding population for the 4th cycle of Chinese fir (Cunninghamia lanceolata (Lamb.) Hook.). Forests. 2023:14:1658. 10.3390/f14081658 PMC9911867 36778690

