# Supplementary material for: Chromosomal evolution and speciation inferred from chromosome-scale genome assemblies in three Cupressaceae species
Source: DNA Res. 2026 Aug 4;33(4):dsag010. doi: 10.1093/dnares/dsag010 (PMC13433000; doi:10.1093/dnares/dsag010)
Supplement: dsag010_Supplementary_Data [file dsag010_supplementary_data.zip › ThreeTreeGenomes-SplFigDNAR.R1.1.pdf]

Supplementary Information

Chromosomal evolution and speciation inferred from chromosome-scale genome assemblies in three Cupressaceae species

Kenta Shirasawa[1*], Yuta B. Aoyagi[1], Kentaro Mishima[2], Hideki Hirakawa[3], Tomonori Hirao[4]

[1]Kazusa DNA Research Institute, Chiba 292-0818, Japan
[2]Kyushu Regional Breeding Office, Forest Tree Breeding Center, Forestry and Forest Products Research Institute, Forest Research and Management Organization, Kumamoto 861-1102, Japan
[3]Faculty of Agriculture, Kyushu University, Fukuoka 819-0395, Japan
[4]Forest Tree Breeding Center, Forestry and Forest Products Research Institute, Forest Research and Management Organization, Ibaraki 319-1301, Japan

*To whom correspondence should be addressed:
Kenta Shirasawa
2-6-7 Kazusa-Kamatari, Kisarazu, Chiba 292-0818, Japan
Tel.: +81-438-52-3935
Fax: +81-438-52-3934
E-mail: shirasaw@kazusa.or.jp

Supplementary Table S1 Mapping populations used for genetic analysis
Supplementary Table S2 Number of marker loci and length of the genetic maps for *Ch. obtusa*
Supplementary Table S3 Number of marker loci and length of the genetic maps for *Cr. japonica*
Supplementary Table S4 Number of marker loci and length of the genetic maps for *Cu. lanceolata*
Supplementary Table S5 Result of GO enrichment analysis for *Cu. lanceolata*

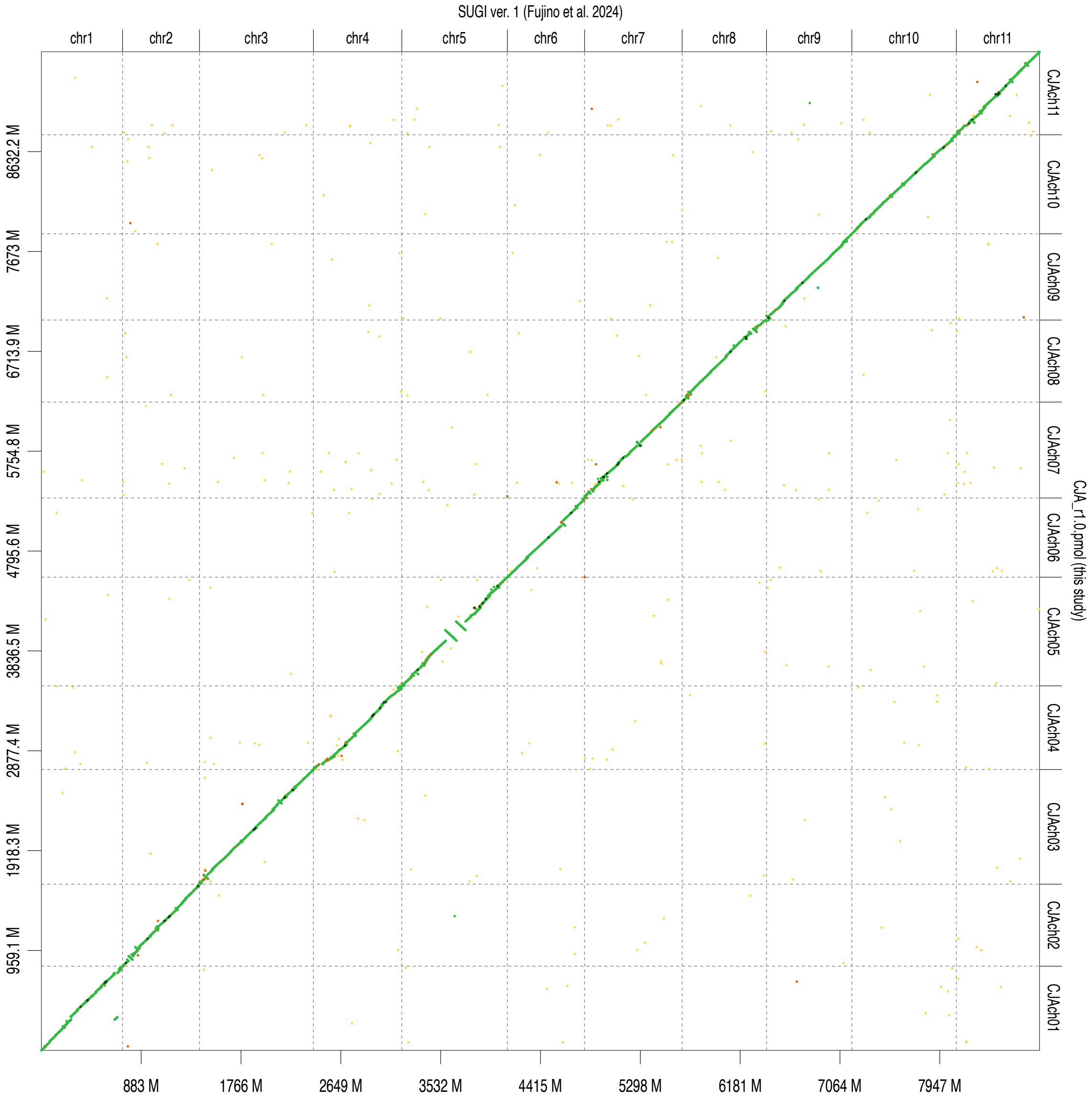

Supplementary Figure S1 Comparative analysis of genome sequences and structures of *Cr. japonica*.

Chromosome numbers are indicated on the top (x-axis) and right (y-axis). Genome sizes (Mb) are shown on the bottom (x-axis) and left (y-axis).

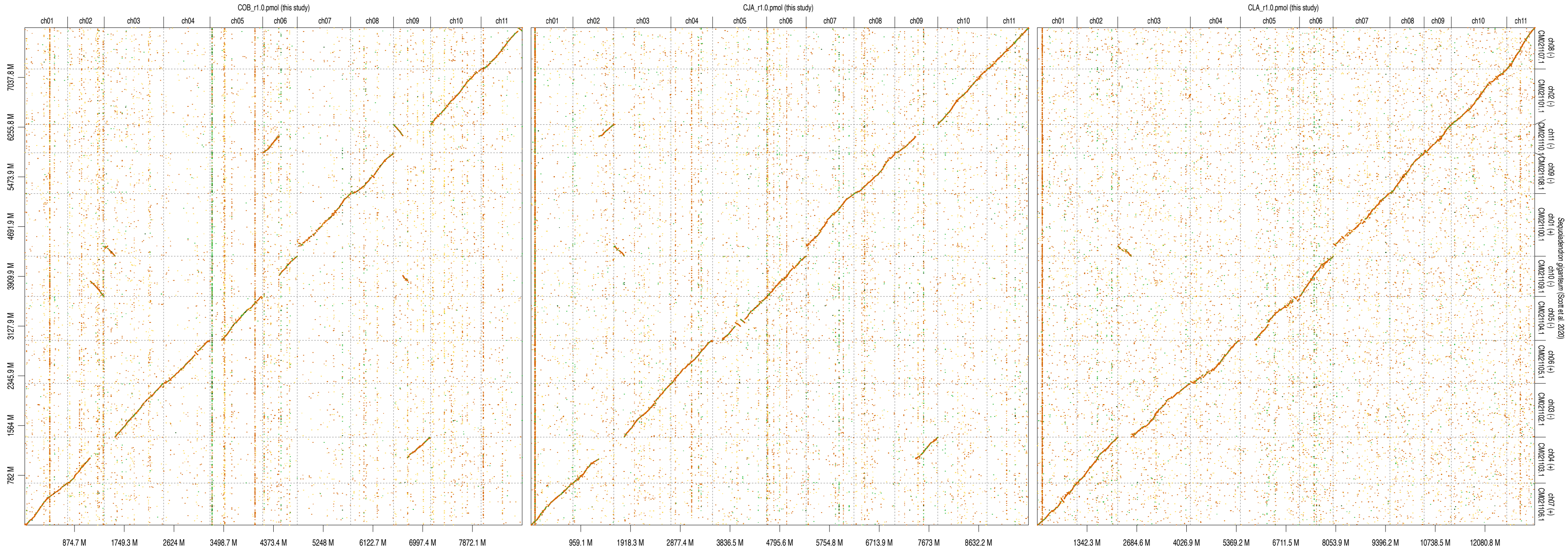

Supplementary Figure S2 Comparative analysis of genome sequences and structures of *S. giganteum* with *Ch. obtusa*, *Cr. japonica*, and *Cu. lanceolata*. Chromosome numbers are indicated on the top (x-axis) and right (y-axis). Genome sizes (Mb) are shown on the bottom (x-axis) and left (y-axis). The order and directions of the *S. giganteum* genome are adjusted as indicated on the right (y-axis).

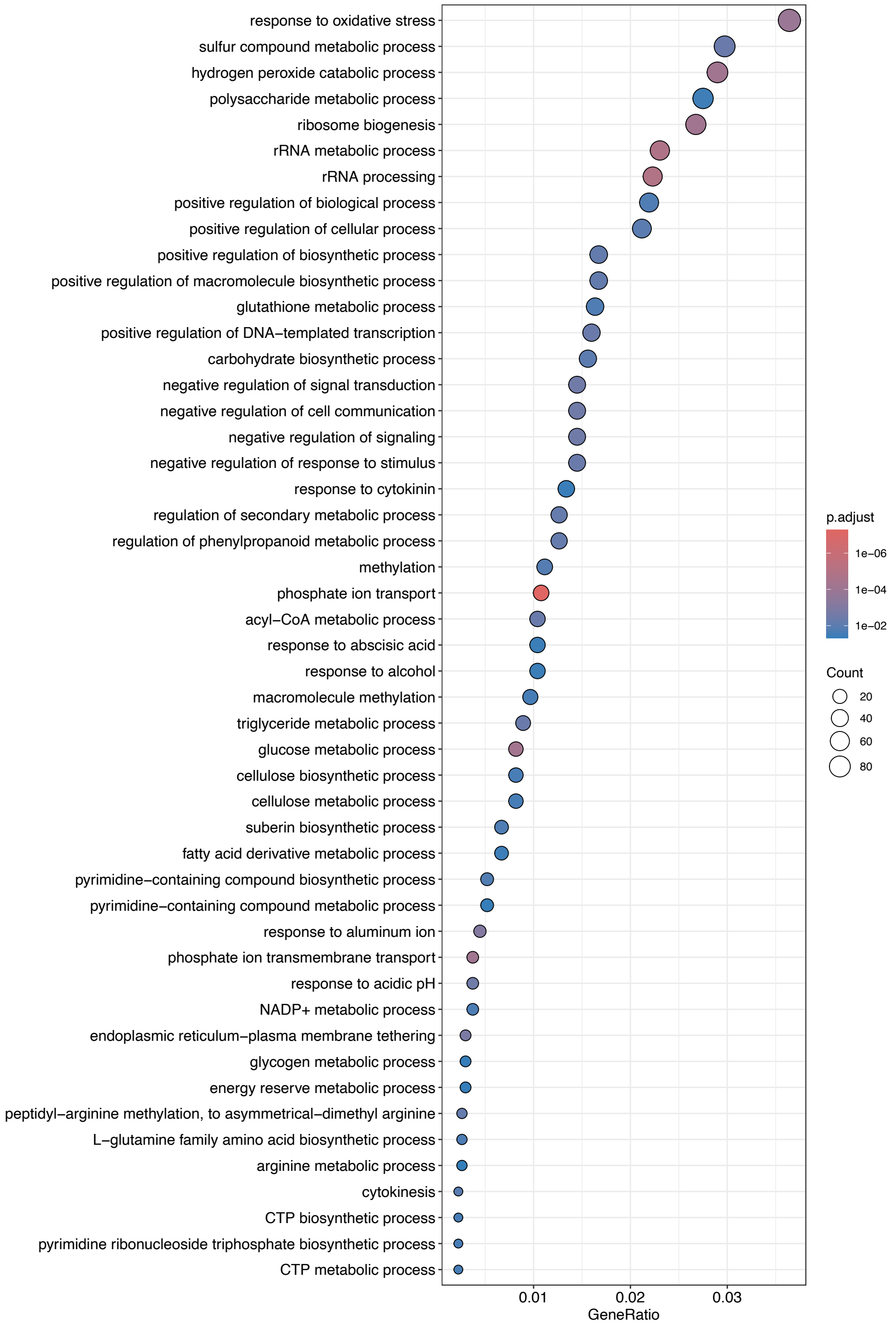

Supplementary Figure S3 Enriched GO terms of paralogous genes in the *Cu. lanceolata* genome.

GO terms with adjusted p-value less than 0.05 are shown. The GO terms are ordered by 'GeneRatio', which indicates the ratio of the number of genes with the corresponding GO term to the total number of target genes. The size of each dot represents 'Count', which indicates the number of target genes with the corresponding GO term.

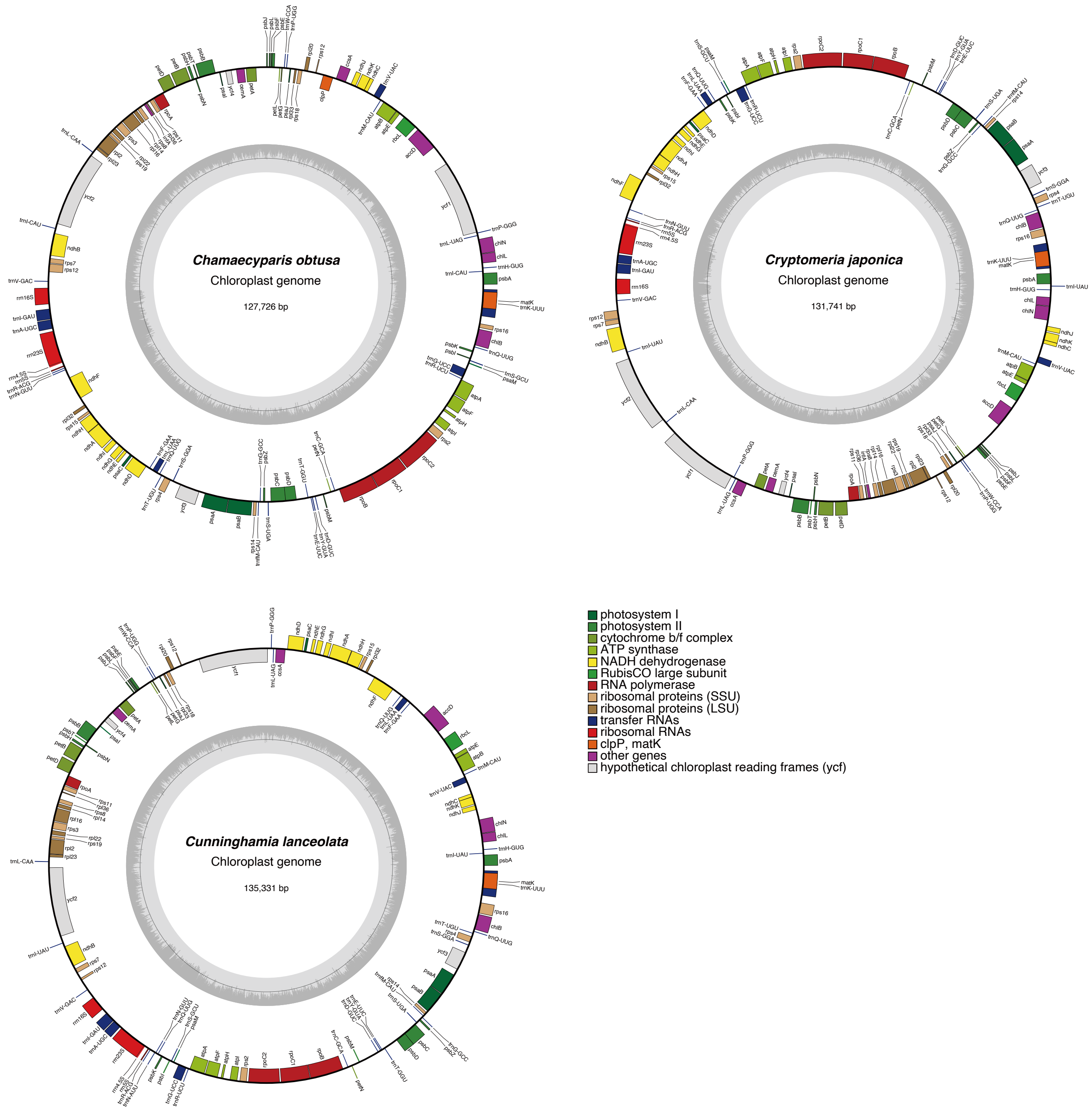

Supplementary Figure S4 Chloroplast genomes of *Ch. obtusa*, *Cr. japonica*, and *Cu. lanceolata*. Boxes on the genome tracks indicate genes. A key to the gene functions is provided in the bottom-right corner.

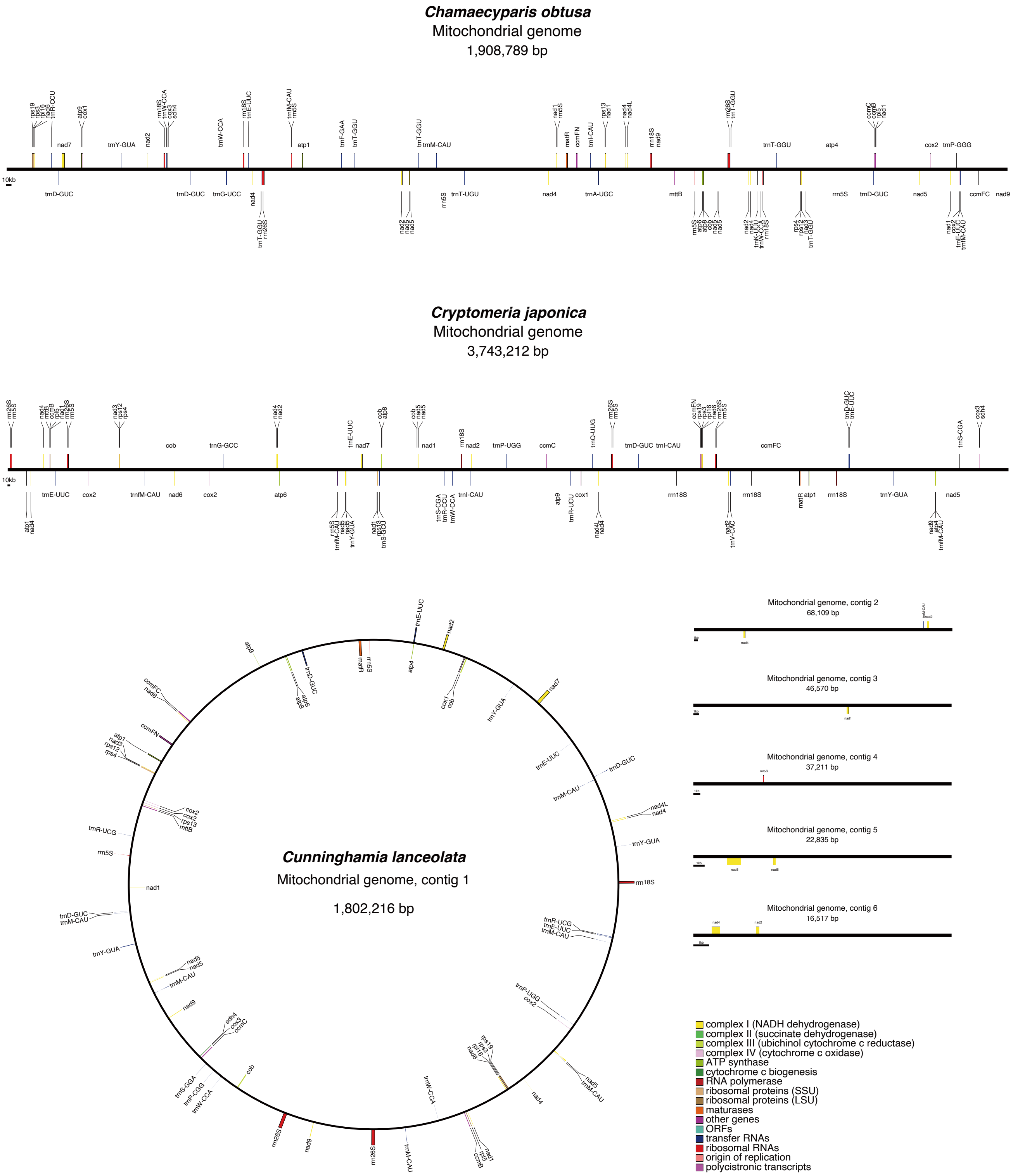

Supplementary Figure S5 Mitochondrial genomes of *Ch. obtusa*, *Cr. japonica*, and *Cu. lanceolata*. Boxes on the genome tracks indicate genes. A key to the gene functions is provided in the bottom-right corner.

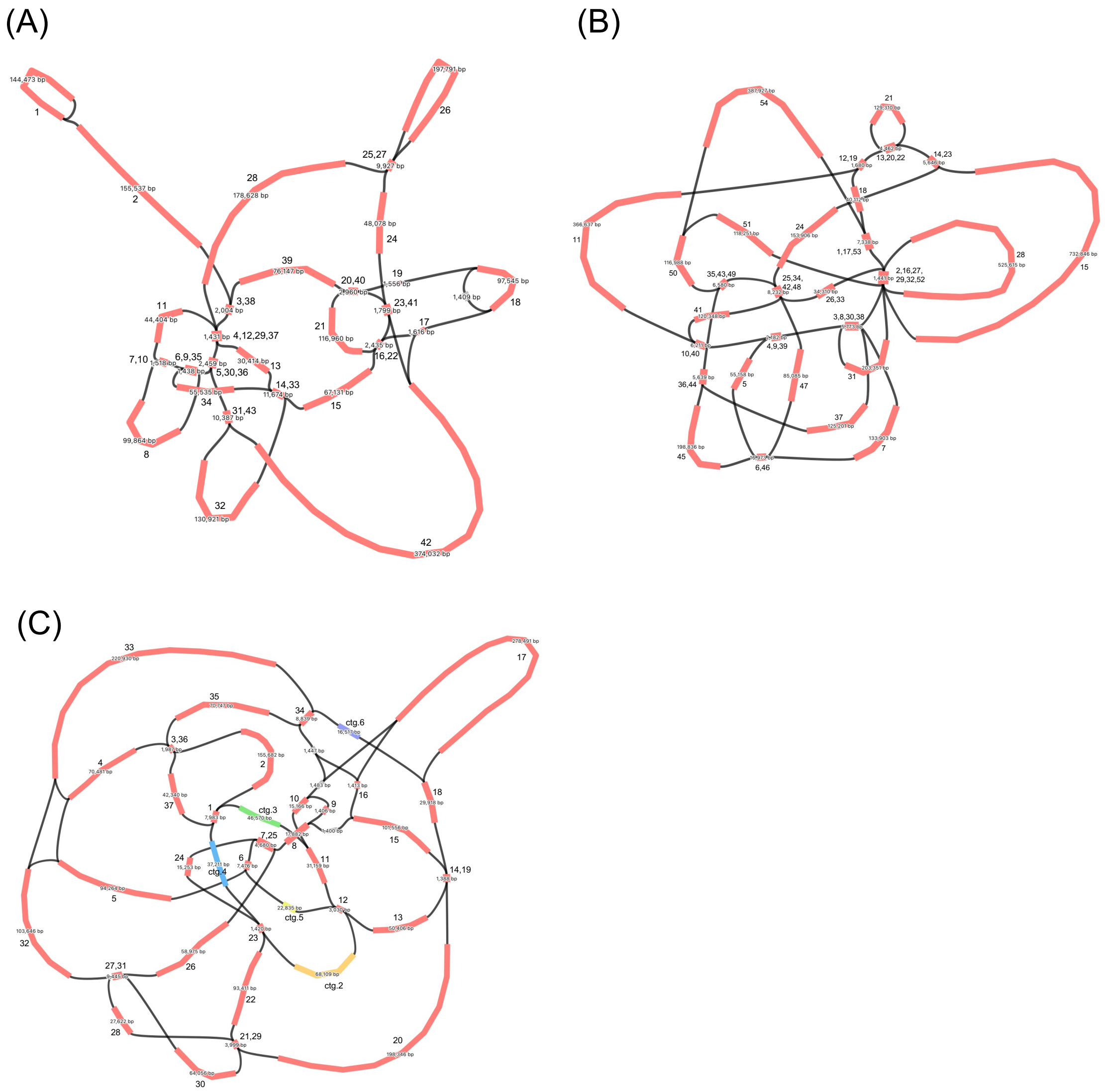

Supplementary Figure S6 Assembly graphs of mitochondrial genomes for (A) *Ch. obtusa*, (B) *Cr. japonica*, and (C) *Cu. lanceolata*.

Rectangles represent nodes, and lines indicate connection of nodes. The length (bp) of each node is shown within the node. The number in each node represents the path in the assembled contigs. Nodes included in the assembled contigs are colored, and different colors represent different contigs. Gray nodes were removed from the assembled contigs.
